# *In vitro* contraceptive activities, molecular docking, molecular dynamics, MM-PBSA, non-covalent interaction and DFT studies of bioactive compounds from *Aegle marmelos*. Linn., leaves

**DOI:** 10.3389/fchem.2023.1096177

**Published:** 2023-01-26

**Authors:** Prasanth Gunasekaran, Yogaswaran Velmurugan, David Stephen Arputharaj, Jose Kavitha Savaridasson, Madhukar Hemamalini, Rajakannan Venkatachalam

**Affiliations:** ^1^ Centre of Advanced Study in Crystallography and Biophysics, University of Madras, Chennai, Tamil Nadu, India; ^2^ Department of Physics, PSG College of Arts and Science, Coimbatore, Tamil Nadu, India; ^3^ Department of Chemistry, Mother Teresa Women’s University, Kodaikanal, Tamil Nadu, India

**Keywords:** *Aegle marmelos*. Linn., natural contraceptives, computer assisted sperm analysis, non-covalent interaction plot, hyaluronidase (HAase)

## Abstract

**Introduction:** Bioactive molecules from natural sources having contraceptive properties were excellent alternatives for modern hormonal contraceptives. Researchers around the world were working on identifying contraceptive leads targeting the male reproductive system rather than the usual female contraceptives. The lack of proper understanding on male contraceptive protein drug targets leads to insufficient evidence on activities of identified contraceptive compounds. The proteins specific to the male reproductive system and involved in sperm-egg fusion will be an excellent drug target to identify the male non-hormonal, reversible contraceptive leads. Inhibiting sperm hyaluronidase activity by natural non-hormonal compounds will lead to reversible and non-hormonal male contraception. The Aegle marmelos Linn. is one such important medicinal plant with valuable phytocompounds, used traditionally as a potential contraceptive measure. The in vivo experiments on leaf extracts of Aegle marmelos. Linn containing terpenes, sterols, and alkaloids shows prominent contraceptive activities. Moreover, this study explores the potential ability of the leaf extract on inhibiting the sperm hyaluronidase action with additional molecular details on the interaction between sperm hyaluronidases and three phytocompounds such as aegeline, marmin, and marminol.

**Material and methods:** The in vitro hyaluronidase inhibition assay and Computer Assisted Sperm Analysis (CASA) were used to evaluate the male contraceptive properties of the Aegle marmelos Linn. leaf extract. To identify the interaction profile of aegeline, marmin, and marmenol on sperm cell hyaluronidases the in-silico methods such as molecular docking, Non-Covalent Interaction analysis, Molecular dynamics, and Molecular Mechanics Poisson Boltzmann Surface Area were used.

**Results and discussion:** The results of in vitro hyaluronidase inhibition assay and Computer Assisted Sperm Analysis shows the inhibition of hyaluronidase enzymatic activity and reduced sperm activities in the presence of leaf extracts. After incubation with leaf extracts for about 30 minutes time intervals show, the motility drops from progressive to non-progressive and ended up with complete immotile in 100 μg/ml concentration of leaf extract. The results of molecular docking, Non-Covalent Interaction analysis, Molecular dynamics, and Molecular mechanics Poisson Boltzmann Surface Area show that the phytocompounds marmin, and aegeline have the potential ability to inhibit sperm hyaluronidase.

## 1 Introduction

Contraceptive measures play an important role in maintaining the reproductive health of an individual, other than the mechanical way of contraception most contraceptive drugs is specific only to women. Also, the available contraceptive drug products mostly target the hormone mechanism of a women individual which creates future side effects for the individual when planning for pregnancy. The area of designing male-specific contraceptive drug leads is still in the developing stage and no proper products were available for use. The place for non-hormonal, reversible male contraceptive agents was still the least explored part in the field of male contraceptive drug design. Most of the drugs such as dimethandrolone undecanoate, 11β-methyl-nortestosterone dodecyl carbonate, 17-methyl-testosterone androgen, and 7α-methyl-19-nortestosterone are testosterone and androgen analogs (Anderson and Baird, 2002; Attardi et al., 2006; Attardi et al., 2011; JillLong et al., 2019). These drug leads are in the clinical trial stage but bear potential side effects on the hormonal mechanism of males. On the other hand, the research on non-hormonal male contraceptives yields many drugs and drug targets such as targeting the retinoic acid synthesizing pathway which was essential for spermatogenesis and bromodomain testis (BRDT) specific protein family, an important protein family necessary for chromatin remodeling during sperm cell development (Anderson and Baird, 2002). For drug lead targeting these pathways lags by the presence of liver damage in alcohol-consumption individuals and the hindrance in designing BRDT inhibitors was their poor specificity to a certain protein in this family. Other than targeting the spermatogenesis mechanism, researchers around the world were working on molecules that inhibit sperm-egg fusion, and acrosome reaction activation mechanism. The proteins such as catsper ion channel proteins present in the membrane of the sperm cell, ABDH2 domain which plays important role in progesterone-based acrosome activation, sperm surface epididymal peptidase involved in calcium influx, and acrosomal proteases and hydrolases were an important class of drug targets which gains interest among researchers nowadays due to their unique nature of specific localization in the sperm cell. But still, there is a need in analysing the effect of contraceptives with proper protein inhibition and sperm cell inhibition methods. To propose a valuable contraceptive lead with the least adverse effects and no effect on the hormonal system will need a proper drug target system and protein target in sperm cells. Proposing sperm-specific hyaluronidases as a potential drug target for designing contraceptive drugs will result in the identification of reversible non-hormonal contraceptive leads. The sperm-specific hyaluronidases are important classes of hydrolases present in the surface, acrosome, and seminal plasma of the human reproductive system. The four isoforms of hyaluronidases such as hyaluronidase-2, hyaluronidase-3, hyaluronidase-4, and hyaluronidase-ph20 are important for the sperm to penetrate the ovum by hydrolyzing hyaluronan layer of an ovum. Inhibiting this enzyme activity hinders sperm cell signaling, sperm cell penetration, and sperm cell movement essential for fertilization. In the field of hyaluronidase inhibitors study, rather than synthetic lead molecules, many natural compounds have an upper hand in hyaluronidase enzyme inhibition with the potential male contraceptive property. The flavonoid content of Justicia gendurussa has the potential ability to inhibit hyaluronidase activity thereby reducing the m penetration and fertilization (Prajogo et al., 2009; Sihabuddin et al., 2011). The research on Terminalia chebula a native plant belonging to India and South East Asia shows the potential ability in inhibiting hyaluronidase activity with *in-vivo* anti-spermatogenic activity (Srivastav et al., 2010). Other than these two plants, compounds and extracts from many plant sources have shown effective male contraceptive results like sperm antimotility properties, anti-spermatogenic activities, anti-spermicidal, and sperm-specific enzyme inhibition activities (Kiran et al., 2014; Tania et al., 2014; Chang et al., 2021; Shunnarah et al., 2021; Verma and Yadav, 2021; Bhatt and Deshpande, 2021; Hifnawy et al., 2021; Long et al., 2019; Norcross et al., 2022; Vijay and Radhey, 2013; Zina et al., 2015). Here we report the male contraceptive ability of *Aegle marmelos* Linn., an important medicinal plant species having a prominent role in the traditional medical practices of India and South East Asia. *A. marmelos*. Linn is such a plant that bears potential and traditional antifertility values with the least research-based evidence (Shahedur and Rashida, 2014). The leaf, bark, and fruit extracts of *A. marmelos* Linn were shown to have an *in vivo* male antifertility effect on rat models (Chauhan et al., 2007; Alka and Meera, 2009). Based on this evidence, our work attempts to evaluate the contraceptive ability of the *A. marmelos*. Linn leaf extract using enzyme inhibition, CASA, and *in silico* methods by proposing sperm-specific hyaluronidases as potential protein targets in male contraceptive drug development.

## 2 Materials and methods

### 2.1 Plant collection and plant extract preparation

The dried, finely macerated form of *A. marmelos*. Linn leaves were collected from Gtee Botanical Extract Private Limited, Perungudi, Chennai, Tamil Nadu, India. The plant was identified and confirmed by the Assistant Professor Dr. A. Selvaraju, Department of Plant Biology and Plant Biotechnology, Gurunanak College of Arts and Science, Velachery, Chennai. About 500 g of fine leaf powder was extracted using ethanol in the Soxhlet extraction apparatus for 20 h of extraction period. The crude extract was separated from the solvent by a Rota evaporator and the solid crude extract was kept at 4-degree cold storage. For the use of *in vitro* hyaluronidase inhibition assay and Computer Assisted Sperm Analysis, the crude solid sample was diluted in the DMSO for 1,00,000 μg/ml, 10,000 μg/ml, 1,000 μg/ml, 100 μg/ml of linear concentrations.

### 2.2 CASA analysis

Computer Assisted Sperm Analysis technique is a modern sperm analysis method used in andrology labs to identify precise and well-characterized parameters of sperm dynamics in infertility treatments. This method combines modern electronic visualizing techniques with sophisticated software programs to evaluate the sperm parameters such as motility, sperm count, sperm DNA fragmentation, and acrosomal status. We adapted the clinical diagnostic technique used in infertility treatment to evaluate the effect of *A. marmelos*. Linn leaf extracts on sperm motility parameters by live imaging of sperm cell velocities. The whole CASA analysis was conducted in Dr. Borus Andro Lab and Research Centre, Vadapalani, Chennai, Tamil Nadu, having proper ethical clearance. The semen samples were collected, evaluated, and incubated in accordance with the regulation of WHO with the help of andrologists (Trevor et al., 2010). The semen samples from healthy individuals were used for the study, and the healthy sperm cell parameters were calculated prior to the experiment according to the WHO recommended values (Jamalan et al., 2016). The prepared *A. marmelos*. Linn leaf extracts (AMLE) with 1 mg/ml, 10,000 μg/ml, 1,000 μg/ml, and control 100 percent DMSO were diluted with equal volumes (20 μl test solution:20 μl semen) of healthy semen and incubated for 30 min in the static incubator. After 30 min the test solution-treated semen samples were carefully placed over cover slides and analyzed for sperm motility changes using a Magnus MX21I LED Binocular microscope. The live changes in the motility of the sperm cells treated with AMLE with respect to untreated sperm cells were monitored using the commercial version of the MMC (MultiMedia Catalog Sperm) software package (Jamalan et al., 2016). The sperm motility was characterized based on parameters such as curvilinear velocity (VCL), straight-line velocity (VSL), average path velocity (VAP), the amplitude of lateral head displacement, beat cross frequency (BCF), and mean angular displacement (MAD). The live images and sperm movement were recorded, processed, and stored in suitable formats. The 100 μg/ml of concentration was used to analyze the morphological and vitality changes in sperm cells due to the AMLE. The vitality test was performed based on the eosin staining method, the 1 drop of 50 μL semen was added with 100 μL of eosin Y solution (2.5 G/L) and incubated for 1–2 min. The smear was produced, dried, and observed under phase contrast Magnus MX21I LED microscope.

### 2.3 *In vitro* bovine testicular hyaluronidase inhibition studies

Hyaluronidase inhibition assay is an important enzyme inhibition assay to evaluate the contraceptive ability of *A. marmelos*. Linn leaf extracts (AMLE). Since the hyaluronidase enzyme used in this assay was bovine testicular hyaluronidase, the results bring maximum probability similar to human sperm hyaluronidase inhibition. The assay was performed based on the modified protocol given in the Sigma Aldrich hyaluronidase inhibition assay manual (Vinata et al., 2005; Ye et al., 2006; Girish et al., 2009; Clifford et al., 2011; Gong et al., 2016). The hyaluronidase enzyme was prepared by dissolving lyophilized enzyme in a buffer containing 20 mM sodium phosphate, 77 mM sodium chloride, and Bovine serum albumin (0.01%) with pH 7.4. The substrate solution of hyaluronic acid was prepared by dissolving solid hyaluronic acid in 300 mM sodium phosphate with 5.4 with 0.03% of hyaluronic acid as the final concentration. About 10 μl of leaf extracts (3 mg/ml, 30 μg/ml, 300 μg/ml, 3,000 μg/ml of final concentration), positive and negative controls were added with 100 μl of hyaluronidase enzyme solution, and preincubated for 10 min at 37 degrees temperature. 100 μL of the hyaluronic acid solution was added to the incubation mixture and kept at 45 degrees temperature for 45 min. The unbound hyaluronic acid molecules were precipitated using 1 mL of precipitation solution containing 24 mM sodium acetate, 79 mM acetic acid, and 1% Bovine Serum Albumin with pH 3.75 and kept at room temperature for 10 min. The unbound hyaluronic acid in each mixture was measured at 600 nm wavelength using UV-VIS JASCO V-630 Spectrophotometer. The absorbance of the mixture without enzyme and mixture without hyaluronic acid were measured initially followed by the measurement of enzyme, substrate, and leaf extract mixture. To measure the percentage of enzyme inhibition the following formula was used

$$Percentage\;of\;Inhibition=\left(1-\frac{Absorbance\;of\;No\;enzyme\;control\;at\;600nm-Absorbance\;of\;leaf\;extract\;mixture\;at\;600nm\;}{Absorbance\;of\;No\;enzyme\;control\;at\;600nm-Absorbance\;of\;No\;inhibitor\;control\;at\;600nm}\right)X\;100.$$

### 2.4 Molecular docking

The molecular docking method is an important computational technique used in the field of drug discovery to identify the molecular interactions of drugs with drug targets. To explore the three important phytocompounds aegeline, marmin, and marmenol known to present in a maximum concentration of all parts of the *A. marmelos*. Linn (Ali and Pervez, 2004), the molecular docking methodology was performed using AutoDock Tools 4.2.1 (Morris et al., 2009). AutoDock tools are important software package available for molecular modeling, molecular docking, and virtual screening. The AutoDock 4 is freely available software for public usage under the GNU general public license. Prior to molecular docking, the structure of phytocompounds was downloaded from the PubChem database [PubChem ID-Aegeline: 15558419, Marmin:6450230, Marmenol (7-(2,6-dihydroxy-7-methoxy-7-methyl-3-octaenyloxy) coumarin): 129847759, Apigenin: 5280443], here we used apigenin as a reference inhibitor of hyaluronidase (Kim et al., 2019). The structure of the compounds was energy minimized using AVAGADRO software using GAFF force field with steepest descent algorithm (Marcus et al., 2012; Avogadro, 2022) and converted into PDBQT file format for molecular docking in AutoDock GUI. The modeled three-dimensional structures of sperm hyaluronidases such as hyaluronidase-2 (HYAL-2), hyaluronidase-3 (HYAL-3), hyaluronidase-4 (HYAL-4), and hyaluronidase-ph20 (HYAL-PH20) were downloaded from the ALPHAFOLD database (Jumper et al., 2021). All the protein structures were protonated using AMBER ff14SB force field and energy was minimized with 100 steepest descent steps, 0.02 Å steepest descent step size, 10 conjugate gradient steps, and 0.02 Å conjugate gradient step size using UCSF CHIMERA software (Pettersen et al., 2004)**.** The receptor pdbqt files were prepared in AutoDock GUI for site-specific molecular docking and the grid size was adjusted to 50 × 50 × 50 with 0.375 Å spacing to cover residues important catalytic activity and substrate binding of human sperm hyaluronidases. The molecular docking was performed with 20 GA runs and the population size of 150, the results are visualized and validated using PyMol (Morris et al., 2009) and LigPlot software (DeLano, 2002).

### 2.5 NCI, DFT analysis

Non-covalent interaction analysis or NCI plot index is an important technique to evaluate the stability of the protein-ligand complex by computing the van der Waals interactions, electrostatic, hydrogen, and hydrophobic interactions. The strong and weak bonding nature of phytocompounds with the hyaluronidases in the binding site were characterized using iso surface plots obtained from the NCI analysis. The reduced density gradient (RDG) plots were used to specify the hydrogen bonding, van der Waals interactions, and steric effects that arise within the hyaluronidase—inhibitor complex. Finally, the QTAIM analysis was used to confirm the strong and weak hydrogen bonding between the amino acids of hyaluronidases and phytocompounds. Multiwfn software version 3.3.1 (Laskowski and Swindells, 2011) was used to produce topological parameter calculation on hydrogen bond stability, Gaussian 09 software (Lu and Chen, 2012) was used to create ligand geometry and the VMD program (Frisch et al., 2010) was used to produce iso surface plots. The binding energies of the interactions of three ligands, aegeline, marmenol, and marmin with the amino acid residues of the proteins, HYAL2, HYAL3, HYAL4, and HYAL-PH20 were well established from the bond topological analysis carried out at (3,-1) bond critical points, by incorporating the Bader’s QTAIM theory using Multiwfn software version 3.3.1. The corresponding topological and energy values were obtained by using the DFT method of B3LYP with the 6-311G basis set and the results are tabulated in Table 4.

### 2.6 Molecular dynamics and MM/PBSA studies

The stability of the protein-ligand complex was analyzed using molecular dynamics methodology, the protein-ligand complexes having the least binding affinity with interactions with active site amino acids were chosen for molecular dynamics. The molecular dynamics simulation of the protein-phytocompound complex was performed in the WEBGRO molecular dynamics web server (https://simlab.uams.edu/index.php) (Bekker et al., 1993; Humphrey et al., 1996; Bjelkmar et al., 2010; Lindorff-Larsen et al., 2010; Abraham et al., 2015) and ligand topology files were generated using the PRODRG web server (Oostenbrink et al., 2004). All the complexes were simulated for a 100 ns time period with 1,000 frames per simulation using GROMOS96 43a1 force field with SPC water model in the triclinic unit cell included in the WEBGRO server. The protein complexes were energy minimized using the steepest descent integrator with 5,000 steps and NVT/NPT equilibration was performed with 300 K temperature, 1 bar pressure in the WEBGRO server. The results such as ligand RMSD, the radius of gyration, solvent accessible surface area, and hydrogen bonds over every frame were analyzed using GROMCAS version 2020.1. MM-PBSA (Molecular Mechanics Poisson Boltzmann Surface Area) calculation method was an important method in analyzing the intermolecular interaction free energies of protein-ligand complexes. The intermolecular interaction free energies such as Van der Wall energy, Electrostatic energy, polar solvation energy, and SASA energy were calculated using the following formula.

$$∆G_{Binding}=∆G_{Complex}–∆G_{Receptor}–{∆G}_{Inhibitor}$$

The MM-PBSA calculations were carried out using g_mmpbsa version 5.1.2 tool (Schüttelkopf and Van Aalten, 2004) which was a free accessible open access package under open-source drug discovery consortium. The MM-PBSA calculations were performed for 50 frames (5 ns, during the simulation period where the ligand RMSD stabled) Kushwaha et al., 2021.

## 3 Results and discussion

### 3.1 Effect of the leaf extracts on sperm motility and morphology and vitality

**FIGURE 1:**
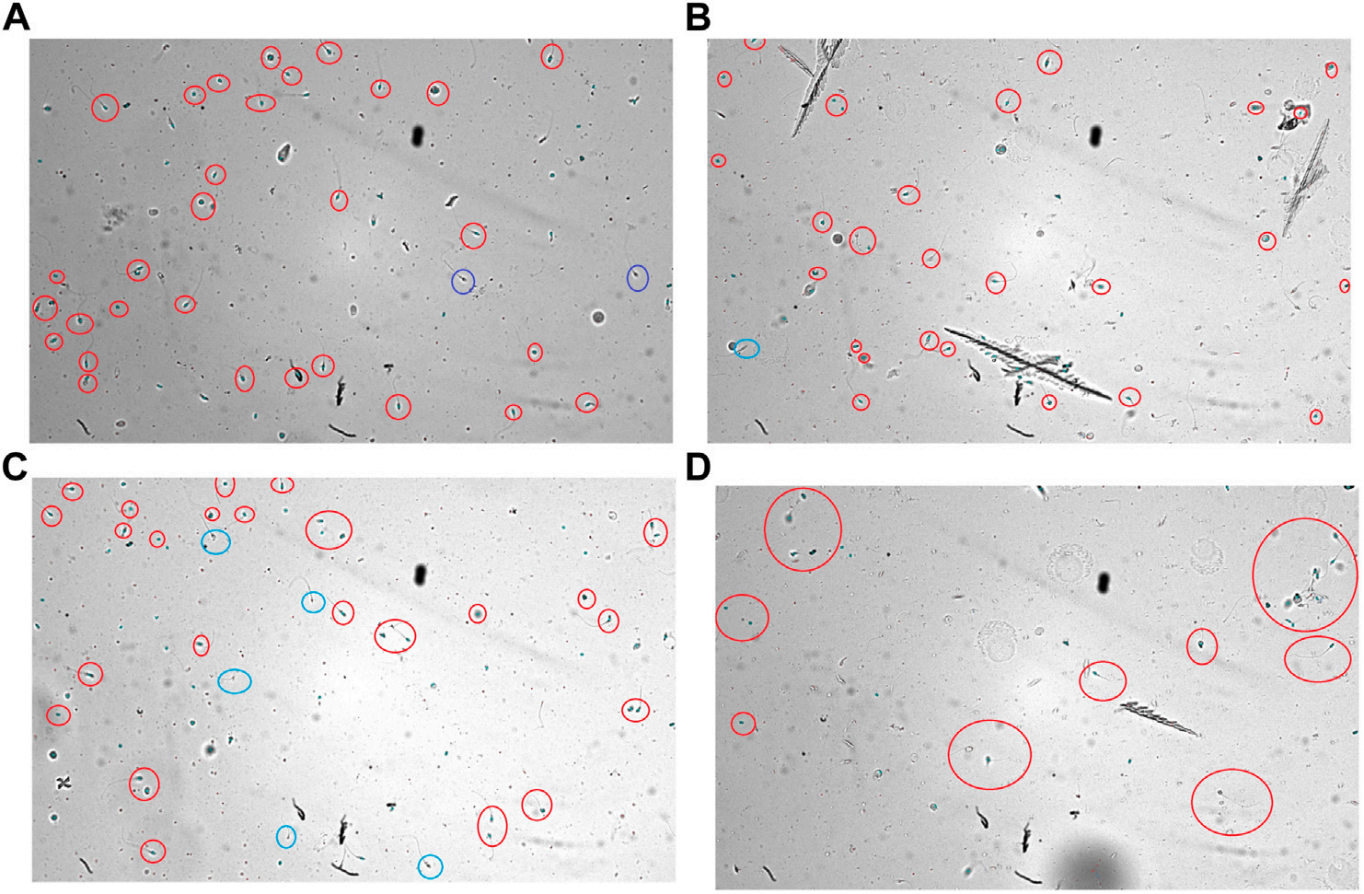
From left to right—Frames recorded from the CASA analysis of AMLE-added sperm cells using MMC software for **(A)**100 μg/ml AMLE **(B)** 1,000 μg/ml AMLE **(C)** 10,000 μg/ml AMLE **(D)** 1,00,000 μg/ml AMLE. The blue circles generated by the software indicate a greater number of immotile cells. To highlight the immotile sperm cells, we manually highlighted the blue marks using red circles. The cells within the blue circle mark indicate a complete loss of sperm cell activity.

**FIGURE 2:**
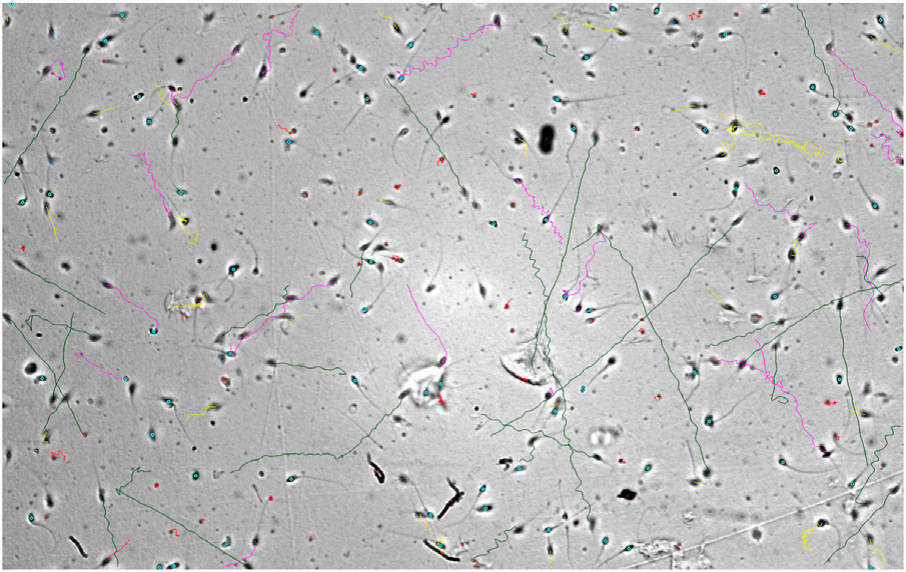
(Right) Frames recorded from the CASA analysis on sperm cells without AMLEusing MMC software. The red, yellow, and black streaks are produced by the MMC softwarewhich recognizes the motility of active and progressive motility.

**FIGURE 3:**
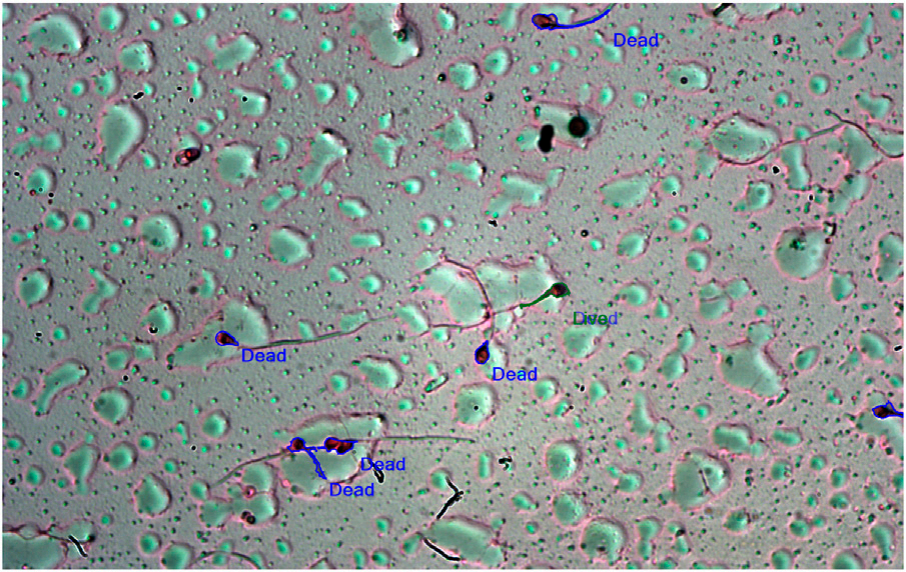
(Left) Image from the vitality test for 100 μg/ml AMLE using the eosin staining method. The blue region indicates the presence of dead sperm cells after the application of 100 μg/ml AMLE. This may be due to the effect of AMLE on acrosomal and cytoskeletal proteins such as hyaluronidases.

**FIGURE 4:**
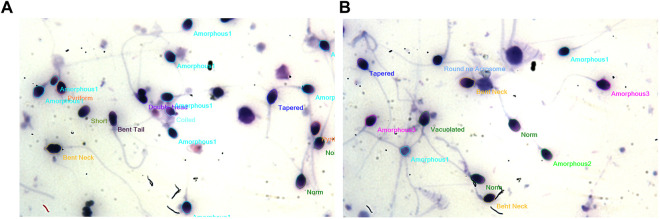
**(A,B)**: Image showing morphological characterization for the effect of 100 μg/ml AMLE on sperm cells showing more amorphous and bent neck cells are observed after incubation.

**FIGURE 5:**
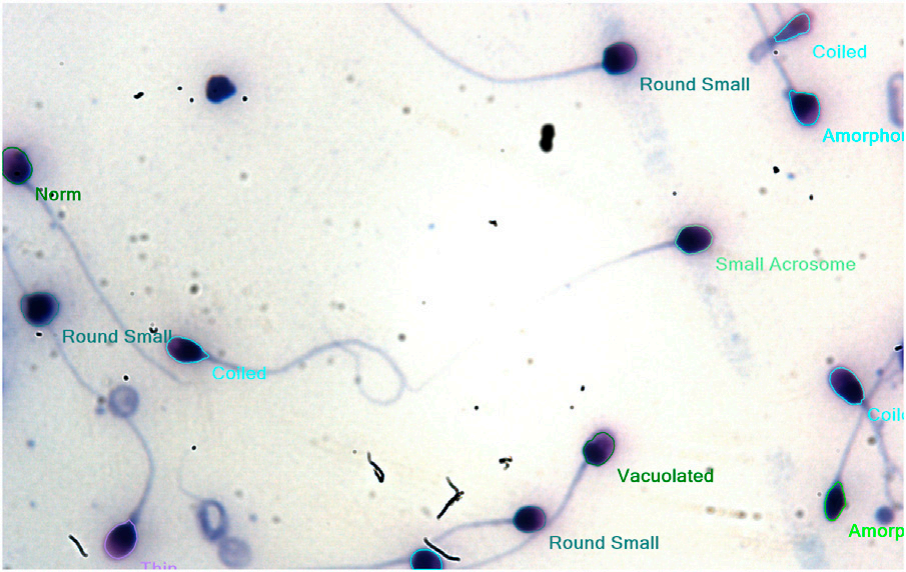
Morphology of healthy sperm cells without AMLE.

The computer-assisted sperm analysis method was used to investigate the contraceptive properties such as affecting the motility of the sperm cells and involvement in remodeling the morphology of sperm cells. To ensure the reversible contraceptive nature of the plant extracts, the vitality of the immotile cells was evaluated. The minimum inhibitory concentration (MIC) for plant extracts of different concentrations diluted in DMSO to inhibit sperm motility was 100 μg/ml. To ensure the effectiveness of the leaf extracts without DMSO the extracts are diluted in a saline medium and the semen samples of healthy individuals were incubated with and without the plant extract for 5 min of incubation time. The results show a considerable decrease in motility parameter values such as VCL, VSL, VAP, BCF, and MAD when compared to semen samples without leaf extracts (Table 1). The sperm motility parameters drop to zero after incubation with the 100, 1,000, 10,000, and 1,00,000 μg/ml of AMLE, and no motile sperm were observed in 1,000 μg/ml (Figures 1–4; Table 2). The sperm morphology modifications and vitality changes were recorded for MIC concentration of 100 μg/ml. The results are compared with the sperm motility parameters incubated without plant extracts and DMSO alone (Table 3). The results from the sperm vitality test show that 100 μg/ml of plant extract produces less amount of spermicidal activity but creates maximum inhibition in motility (Figure 3). This shows that the plant extract produces a defect in the cytoskeletal arrangement of spermatozoa and creates more morphological deformation (Table 3; Figures 4A, B, 5).

**TABLE 1:** Variation in sperm motility parameters in the presence and absence of AMLE diluted in saline.

	VCL (μm)		VSL (μm)		VAP (μm)		ALH (μm)		BCF (per second)		LIN		STR		WOB		MAD	
	C	T	C	T	C	T	C	T	C	T	C	T	C	T	C	T	C	T
Class A	51	33	39	26	39	9.4	1	0.41	2.9	0.67	0.78	0.83	0.84	0.69	0.72	0.23	38	22
Class B	21	18	13	9.1	13	11	0.78	0.82	4	4.4	0.65	0.5	0.79	0.75	0.57	0.62	54	67
Class C	27	8.3	14	3.5	14	4.5	0.61	0.45	4	4.9	0.51	0.52	0.41	0.61	0.42	0.6	51	74
Class D	7	4.2	0.94	0.87	0.94	2.1	0.4	0.35	5.4	4.5	0.16	0.26	0.27	0.41	0.64	0.54	94	108

VSL, straight-line (rectilinear) velocity; VCL, curvilinear velocity. A measure of cell vigor; VAP, average path velocity; ALH, the amplitude of lateral head displacement; LIN, linearity; The linearity of a curvilinear path, VSL/VCL; STR, straightness. Linearity of the average path, VSL/VAP; WOB, wobble. VAP/VCL; BCF, beat-cross frequency. The average rate at which the curvilinear path crosses the average path. MAD, mean angular displacement; C- Control (Sperm cell parameters without extract). *t*-Test [Sperm Cell parameters with AMLE 0.1 g diluted in sperm with a 1(AMLE):10 (sperm) ratio].

**TABLE 2:** Effect of different concentrations of AMLE on sperm motility after 30 min of incubation.

Sample concentration	Total cells million per ml	Immotile cells million per ml	Percentage of immotile after 30 min of incubation
No Inhibitor	13.4	8.11	60.8
DMSO	11.6	9.77	83.9
100 μg AMLE	12.8	12.6	98.5
1,000 μg AMLE	24.4	24.4	100
1,000 μg AMLE	14.3	14.3	100
1,00,000 μg AMLE	12.8	12.8	100

**TABLE 3:** Sperm vitality parameters after 10 min incubation with 100 μg/ml of AMLE.

Defects	Relative to the total number of analyzed sperms %	Relative to abnormal sperms, %
Head defects		
Tapered	4.6	4.9
Pyriform	6.2	6.6
Round, no acrosome	1.5	1.6
Round, small	0	0
Amorphous, type 1	43.1	45.9
Amorphous, type 2	1.5	1.6
Amorphous, type 3	3.1	3.3
Vacuolated	1.5	1.6
Small acrosome	1.5	1.6
Double head	1.5	1.6
Pinhead	18.5	19.7
Neck defects		
Bent neck	4.6	4.9
Asymmetrical	0	0
Thick insertion	0	0
Thin	0	0
Tail defects		
Short	3.1	3.3
Bent	1.5	1.6
Coiled	1.5	1.6
ERC	0	0

### 3.2 Hyaluronidase inhibition assay

The results of the hyaluronidase inhibition assay using bovine testicular hyaluronidase show that complete enzyme inhibition was achieved for 3000 μg/ml of leaf extracts. The enzyme inhibition begins at 3 μg/ml (26%) but there was no increase in enzyme inhibition percentage for 30 μg/ml (26%). The increase in inhibition starts at 300 μg/mL of an extract with 34 percent of enzyme inhibition. The results show that the concentration of plant extracts required for the minimum inhibition was 30 μg/ml and 3,000 μg/ml for complete inhibition of enzyme activity (Table 4).

**TABLE 4:** Results of Hyaluronidase inhibition assay for AMLE.

Sample concentration	Mean Abs_600_	Percentage of inhibition
No enzyme	1.0902	—
No Inhibitor	0.61323	—
3,000 μg AMLE	1.0868	99.28
300 μg AMLE	0.7798	34
30 μg AMLE	0.7407	26
3 μg AMLE	0.7377	26

### 3.3 Molecular docking results

#### 3.3.1 Inhibitor binding site modeling for sperm hyaluronidases

The aegeline, marmin, and marmenol are docked in the binding site surrounded by catalytic and substrate binding residues of human sperm hyaluronidases. The binding site residues were chosen based on literature studies and sequence alignment of human sperm hyaluronidases with human hyaluronidase-1 involved in tumor angiogenesis (Robert and Mark, 2006; Chao et al., 2007; Kumari et al., 2014; Jung, 2020). The residues important for HYAL-2 enzymatic activity were Glu 135, Asp 133, Tyr 206, Tyr 253, Trp 327, Trp 328. The hyaluronan binding cleft of HYAL-3 is lined with residues such as Glu 129, Asp 127, Tyr 202, Tyr 246, and Trp 319. The residues such as Glu 147, Asp 145, Tyr 218, Cys 263, and Trp 339 were important residues of HYAL-4 for their substrate binding and activity. The HYAL-PH20 hyaluronan binding cleft is lined with amino acids such as Glu 148, Asp 146, Tyr 219, Tyr 264, and Trp 339. The above-mentioned residues in the sperm cell hyaluronidases are the target residues for the inhibitor interactions, the molecular docking results show aegeline, marmin, and marmenol interact through hydrogen and hydrophobic interactions within the substrate binding cleft.

#### 3.3.2 Molecular interactions of aegeline, marmin, and marmenol with human sperm hyaluronidases

**FIGURE 6:**
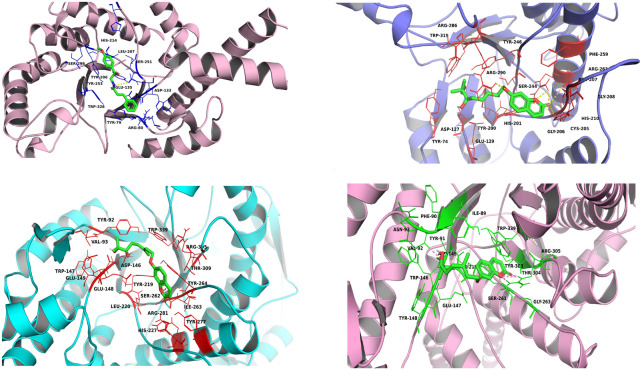
**(A–D)**: (Clockwise) Molecular interaction of aegeline with HYAL-2 marmenol with HYAL-3, marmin with HYAL-4, marmin with HYALPH20 in the substrate and active site region.

The docking results of aegeline, marmin and marmenol with hyaluronidase-2 show the aegeline-HYAL-2 complex has the least binding affinity of −7.0 kcal/mol with the highest number of hydrogen and hydrophobic interaction in the active site cleft. The aegeline interacts with active site residue Glu 135 through hydrogen bond interaction of 2.8 Å and interacts through hydrophobic interaction with substrate binding residues Asp 133, Tyr 206, Tyr 253, Trp 328 (Figure 6). The results from the molecular docking of hyaluronidase-3 with aegeline, marmin, and marmenol confirms that marmenol shows important hydrogen and hydrophobic interactions in the substrate-binding cleft of hyaluronidase-3. Most of the hydrogen bond interactions of marmenol were only with residues not involved in substrate binding and residues such as Asp 127, Glu 129, Trp 319, and Tyr 246 which involves in substrate adoption create hydrophobic interactions with the marmenol. The interactions of hyaluronidase-4 with marmin were found to be more favorable when compared with aegeline and marmenol. The binding affinity of the hyaluronidase-4—marmin complex was at −6.6 kcal/mol where marmin makes hydrogen bonds with Val-92 and Arg-305. Analyzing the hydrophobic interaction pattern marmin binds deeply in the active site cleft region lined with Phe-90, Ile 89, Tyr 218, Tyr 303, Ser 261, Thr 304, Trp 339, Gly 263, Asp 145, Trp 146, Tyr 91, Glu 147, Asn 93, Tyr 148. Among all interaction patterns of human sperm hyaluronidase with the marmin, marmenol, and aegeline, the interactions of hyaluronidase-ph20 with marmin were found to be more stable. The hyaluronidase-ph20—marmin complex was stabilized by the presence of four hydrogen bonds with Asp 146, Glu 148 and hydrophobic interactions with residues such as Glu 149, Tyr 92, Trp 339, Tyr 264, Thr 309, Tyr 277, Leu 220, Ser 262, Arg 281, Tyr 219. The interaction of marmin covers all important amino acids necessary for the hyaluronidase-ph20 activity (Table 5). The To evaluate the complete information about the interaction pattern other than polar and non-polar interactions for the above-mentioned complexes we performed NCI analysis.

**TABLE 5:** Hydrogen and Hydrophobic interaction data analyzed using LigPlot from the molecular docking results.

Compounds	Binding affinity kcal/mol	Hydrogen bonds	Hydrogen bond distance (Å)	Hydrophobic interactions
Hyal-2-Aegeline	−7.0	Glu 135	3.00	Arg80, Tyr 79, Asp 133, Trp 328, Tyr 206, Ser 251, Tyr 253, Ser 299
Hyal-2-Marmin	−6.5	Glu 135	2.87	Asp 136, Trp 134, Tyr 206, Tyr 253, Trp 328, Tyr 79
		Asp 133	3.19	
		Asp 133	2.99	
		Arg 80	2.99	
Hyal-2-Marmenol	−6.1	Arg 295	2.80	Ser 299, Glu 135, Asp 133, Tyr 79, Trp 328, Asp 330
		Tyr 206	3.13	
		Arg 80	3.17	
Hyal-3-Marmenol	−6.5	Arg 290	2.80	Trp 319, Asp 127, Tyr 246, Arg 286, Asn 207, His 201, Gly 206, Gly 208
		Glu 129	2.70	
		Ser 244	2.89	
		Tyr 200	2.81	
		Arg 263	2.82	
		Arg 263	3.12	
		Arg 263	2.99	
Hyal-3-Marmin	−6.3	Tyr 202	2.92	Glu 129, Ser 244, His 201, Tyr 246, Ala 204, Arg 263, Phe 259, Gly 208, Arg 142
Hyal-3-Aegeline	−5.9	—	—	Tyr 74, Asp 127, Glu 129, Tyr 200, Trp 319, Tyr 246, Arg 290, His 201, Arg 290, Ser 244, Arg 263, Gly 206, Gly 208 (
Hyal-4-Marmin	−6.6	Asp 145	2.78	Trp 339, Tyr 303, Tyr 218, Tyr 91, Tyr 148, Glu 147
		Val 92	3.03	
		Arg 305	2.78	
		Arg 305	3.07	
Hyal-4-Marmenol	−6.0	Arg 305	2.03	Arg 309, Tyr 303, Trp 339, Glu 147, Tyr 91, Tyr 218, Asp 145
		Arg 305	2.80	
		Arg 305	2.98	
		Asp 341	2.91	
Hyal-4-Aegeline	−5.9	Arg 309	2.98	Asp 145, Arg 305, Lys 160, Glu 147, Leu 219, Tyr 218, Trp 339
		Arg 150	3.03	
Hyal-ph20-Marmin	−7.3	Asp 146	2.96	Trp 339, Tyr 219, Tyr 264, Ser 262, Thr 309, Tyr 92, Tyr 277, Glu 149, Leu 220, Arg 281
		Asp 146	3.05	
		Glu 148	4.75	
Hyal-ph20-Aegeline	−6.9	Glu 148	4.15	Asp 146, Tyr 92, Tyr 219, Tyr 264, Ser 262, Leu 220, Thr 309, Tyr 277
Hyal-ph20-Marmenol	−6.5	Asp 146	2.95	Tyr 219, Ser 262, Tyr 264, Tyr 277, Thr 309
		Glu 148	4.90	
		Asn 266	2.97	

### 3.4 NCI analysis

**FIGURE 7:**
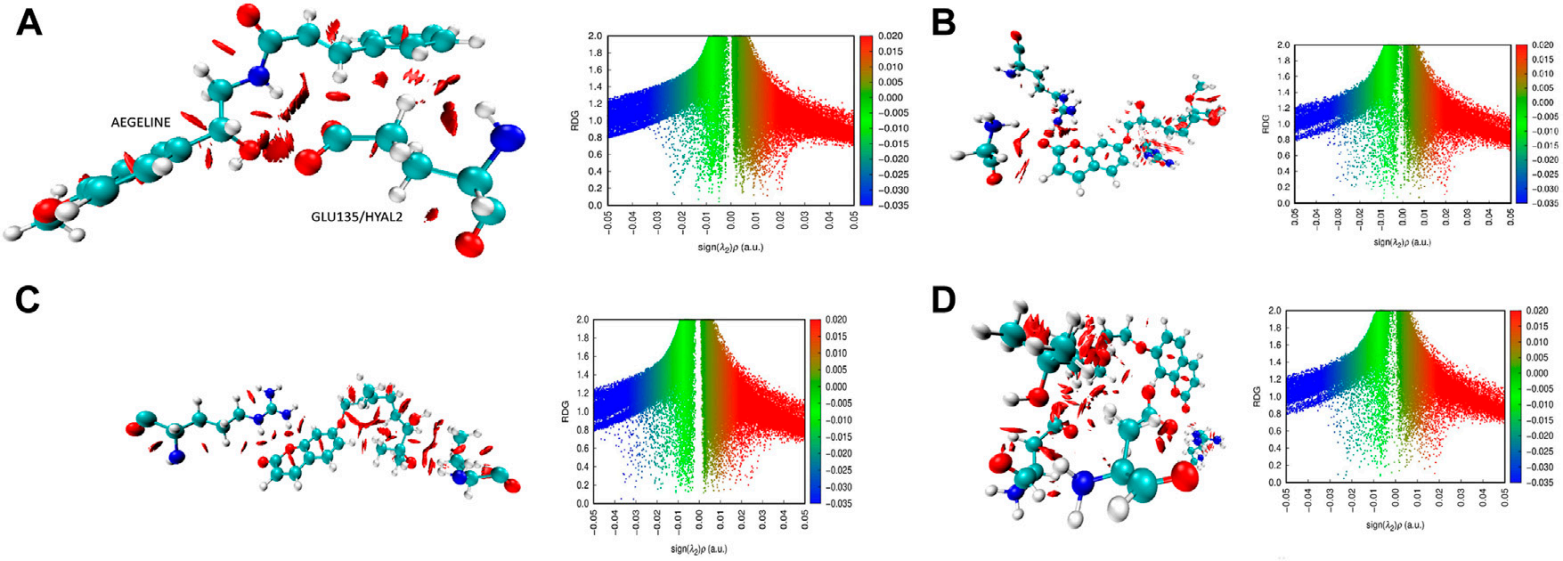
**(A)**: NCI iso surface plot for the HYAL-2-aegeline, **(B)**: NCI iso surface plot for the HYAL3-marmenol, the presence of blue regions within the plot denotes the existence of strong interactions like hydrogen bonds. The presence of red regions in the plot shows thepresence of steric effects due to strong repulsion interactions. The green regions denoted thepresence of weak bonding such as van der Waal interactions. **(C)**: NCI iso surface plotand RDG plot for the HYAL4-marmin, **(D)**: NCI iso surface plot for the HYAL-PH20- marmin.

Exploring the van der Waals interaction energy, the average binding energy of Hyaluronidase-aegeline, marmin, and marmenol complexes reveals there was a string interaction of marmin with the Hyal4 enzyme. From Table 6, it is clearly evident that the ligand Marmin makes strong interaction with ARG305 (O62/HYAL4...H33/ARG305), VAL92 (O65/HYAL4...H8/VAL92) of HYAL4 and the corresponding energy values were −37.54 Kj/mol and −23.23 Kj/mol respectively. It is also found that the interaction of Marmin with HYAL-PH20 is relatively weak with an average binding energy of −7.90 Kj/mol. Similar strength of interaction (O86/HYAL3...H27/ARG263 and O87/HYAL3...H25/ARG263) is found for the ligand Marmenol with amino acid ARG 263 of HYAL3 with the energy values −31.61 Kj/mol and −22.22 kJ/mol respectively. Among the four protein-ligand complexes, the HYAL2-Aegeline complex was the least stable established from the weak hydrogen bonding interaction, HYAL2/H42...O9/GLU135 with an energy value of −7.48 Kj/mol. The interaction spectrum of three ligands with their respective enzymes was further investigated from non-covalent interaction (NCI) analysis. The reduced density gradient (RDG) plots in which blue, green, and red regions reveal the hydrogen bonding, van der Waals, and steric effect interactions between protein and ligand. The strong binding affinity of Marmin towards the active site of HYAL4 protein was clearly visualized from NCI surface and RDG scattering plots (Figure 7).

**TABLE 6:** Bond topological parameters of H-bond interactions calculated at bcp of complexes.

Complex	Bond	ρ [e/Å^3^]	Δ^2^ ρ [e/Å^5^]	V(r) (Kj/mol)	G(r) (Kj/mol)	H(r) (Kj/mol)	E (Kj/mol)
HYAL2-AEGELINE	HYAL2/H42...O9/GLU135	0.06	0.70	−14.97	17.05	2.08	−7.49
HYAL3-Marmenol	O87/HYAL3...H9/GLY206	0.10	0.95	−27.52	26.67	−0.86	−13.76
	O85/HYAL3...H51/ARG290	0.11	1.44	−35.25	37.30	2.05	−17.62
	O86/HYAL3...H27/ARG263	0.22	2.23	−63.23	62.01	−1.23	−31.62
	O87/HYAL3...H25/ARG263	0.16	1.32	−44.44	40.14	−4.30	−22.22
HYAL4-Marmin	O63/HYAL4...H30/ARG305	0.14	1.22	−38.47	35.85	−2.62	−19.24
	O62/HYAL4...H33/ARG305	0.25	2.78	−75.08	75.36	0.28	−37.54
	O65/HYAL4...H8/VAL92	0.15	1.54	−46.47	44.21	−2.26	−23.23
HYALPH20-Marmin	H84/HYAL20PH...O4/ASP146	0.05	0.69	−14.73	16.73	2.00	−7.36
	O80/HYAL20PH...H48/ARG281	0.04	0.53	−12.26	13.36	1.10	−6.13
	H83/HYAL20PH...O23/GLU148	0.07	0.78	−20.42	20.80	0.38	−10.21

### 3.5 Molecular dynamics and MM-PBSA studies

**FIGURE 8:**
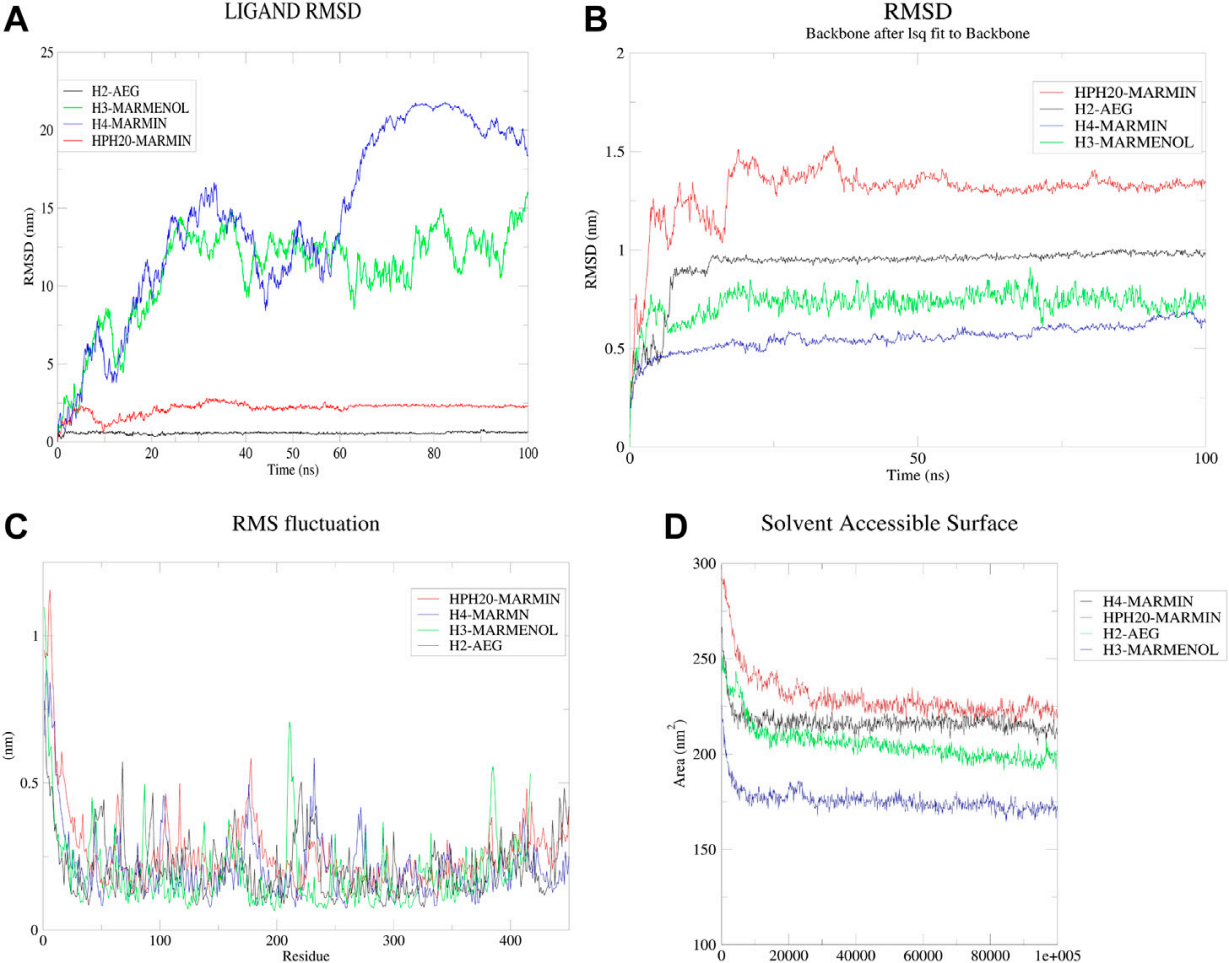
**(A–D)** (clockwise): Molecular dynamics results such as Ligand RMSD, protein backbone RMSD, RMSF and SAS for the protein-ligand complexes.

Based on the results of molecular docking the molecular dynamics for hylaluronidase-2- aegeline, hyaluronidase-3-marmenol, hyaluronidase-4-marmin, and hyaluronidase-ph20- marmin complexes were simulated for 100 ns (Figure 8). Analyzing the ligand RMSD, backbone RMSD, RMSF values of simulated complexes the marmin-hyaluronidase 4, marmin-hyaluronidase ph20, and aegeline-hyaluronidase 2 has stable structure throughout the simulation. The complex RMSD analysis of four protein-ligand complexes shows only hyaluronidase 2-aegeline and hyaluronidase ph20-marmin were so stable. The complex RMSD graph of HYAL2-aegeline and HYALPH20-marmin was stabilized after 25–50 ns. The backbone RMSD results of the molecular simulation were well understood by the backbone RMSD graph of four protein-ligand complexes. No more backbone deviation was observed in the complexes HYAL2-aegeline, HYAL4-marmin, and HYALPH20-marmin after 30 ns of simulation, but for HYAL3-marmenol the structure was stabilized only after 70 ns on reaching 7.5 Å of backbone RMSD. The hydrogen bond profile from the molecular simulations was well characterized with the results from the molecular docking, the hyaluronidase 2- aegeline complex was stabilized by the presence of one to three intermolecular hydrogen bonds. But over the simulation, the graph shows one hydrogen bond was very stable, which must be the bond with aegeline and Glu 135, an active site residue of hyaluronidase-2. For the HYAL3, HYAL4, and HYALPH20 complexes the simulation results show a maximum of four hydrogen bonds for HYAL3-marmenol, HYAL4-marmin complex and two hydrogen bonds between marmin and HYALPH20. The RMSF graph of four protein-ligand complexes was very with least fluctuation throughout the molecular simulation, only HYAL3-marmenol complex has more fluctuation in the region of 200–210 residues. To understand the free energy stabilization of protein-inhibitor complexes we use MM-PBSA method calculation for 50 frames of all four protein-inhibitor complexes. The HYAL2 was stable at the simulation time of 60–65 ns, HYAL4 reaches stability only between 75 and 80 ns, and HPH20 was completely stable after 50 ns. About 50 frames within this simulation time were used in MM-PBSA calculation. But for HYAL3 the complex was stable so we perform MM-PBSA calculation at end of the simulation. The results of MM-PBSA show among the four complexes, the hyaluronidase-4-marmin complex has the least binding energy of −194.916 ± 10.495 kJ/mol, Van der Waal energy of −233.510 ± 10.370 kJ/mol, electrostatic energy of −11.918 ± 4.705 kJ/mol and SASA energy −17.257 ± 0.840 kJ/mol (Table 7).

**TABLE 7:** Results of free energy calculation using MM-PBSA calculation of Hyaluronidase-phytocompounds complexes.

Intermolecular energy	Hyaluronidase-2-aegeline	Hyaluronidase-3-marmenol	Hyaluronidase-4-marmin	Hyaluronidase-PH20-marmin
Van der Waal energy kJ/mol	−186.988 ± 9.634	−188.460 ± 9.733	−233.510 ± 10.370	−127.971 ± 9.989
Electrostatic energy kJ/mol	−0.307 ± 2.469	−10.955 ± 4.017	−11.918 ± 4.705	−11.073 ± 3.352
Polar solvation energy kJ/mol	47.094 ± 7.179	48.423 ± 5.631	67.769 ± 7.390	29.893 ± 11.353
SASA energy kJ/mol	−16.284 ± 0.809	−15.134 ± 0.907	−17.257 ± 0.840	−10.250 ± 1.423
Binding energy kJ/mol	−155.871 ± 11.680	−166.126 ± 10.652	−194.916 ± 10.495	−119.400 ± 12.691

#### 3.5.1 Discussion

The results of CASA studies on the effect of *A. marmelos* Linn., leaf extract show the leaf extract creates temporary inactivation of sperm activity and changes in head morphology. The changes in the motility of the sperm cells due to leaf extracts were due to changes produced in the cytoskeletal and sperm head structure. The hyaluronidase inhibition assay also supports the CASA results of plant extracts. The results of molecular docking with sperm hyaluronidase show the compounds aegeline and marmin have a high capability of inhibiting the enzyme activity when compared to apigenin a natural hyaluronidase inhibitor. The molecular dynamics results such as complex RMSD, backbone RMSD and RMSF values confirm that the aegeline, marmin complex has a high probability to produce enzyme inhibition. The NCI and MM-PBSA results are key results in establishing the potential ability of marmin and aegeline to bind with hyaluronidase isoforms. Especially, marmin shows prominent results in all four *insilico* characterizations, where the marmin-HYAL4 complex shows the least binding values among all in MM-PBSA results and this result supports the NCI topological parameter results where the marmin-HYAL4 has strong interactions. The above results are based on the studies targeting the hyaluronidase enzyme in the active and substrate-binding sites, which ensures that the results are more reliable in the terms of the potential ability of AMLE and its compounds in enzyme-inhibiting activity. Further, future studies to understand the effect of marmin and aegeline in sperm cells will be carried out.

## 4 Conclusion

The male contraceptive effect for leaf extracts of *A. marmelos*. Linn was analyzed and the results from the CASA analysis of motility parameters, vitality, and morphological characterization show leaf extracts of *A. marmelos*. Linn has the potential ability in inhibiting the sperm vital parameter necessary for male fertility. The sperm vitality results confirm the *A. marmelos*. Linn leaves and their compounds have reversible contraceptive effects on sperm cell activity. The hyaluronidase inhibition assay and *in silico* analysis such as molecular docking, molecular dynamics, MM-PBSA, and NCI analysis confirms that the marmin and aegeline have the potential ability to inhibit the enzyme activity. The results also implicate sperm hyaluronidases as a potential target for male contraceptive drug development. Further research in the future will focus on analyzing the effect of these compounds on hyaluronidase and other important enzymes essential for sperm activity by understanding the protein-ligand dynamics.

## Data Availability

The original contributions presented in the study are included in the article/supplementary material, further inquiries can be directed to the corresponding author.
